# Efficacy and Safety of the Treatment of Chronic Hepatitis C with Sofosbuvir/Ledipasvir in Children Aged 5 to 10 Years with Comorbidities—A Brief Report

**DOI:** 10.3390/idr14040061

**Published:** 2022-08-03

**Authors:** Maria Pokorska-Śpiewak, Anna Dobrzeniecka, Agnieszka Ogrodnik

**Affiliations:** 1 Department of Children’s Infectious Diseases, Medical University of Warsaw, 01-201 Warsaw, Poland; 2 Regional Hospital of Infectious Diseases in Warsaw, 01-201 Warsaw, Poland; adobrzeniecka@zakazny.pl; 3 Pomeranian Center for Infectious Diseases and Tuberculosis, 80-214 Gdańsk, Poland; aogrodnik@szpitalepomorskie.eu

**Keywords:** children, chronic kidney disease, direct acting antiviral, hepatitis C virus, sofosbuvir/ledipasvir

## Abstract

The efficacy and safety of 12 weeks of therapy with sofosbuvir/ledipasvir in three patients aged 5–10 years are presented. All three children suffered from comorbidities, including chronic kidney disease in two. All participants achieved a sustained virologic response 12 weeks after the end of treatment. No adverse effects were reported during or after the treatment, and the compliance was good. Decisions on starting treatment in children below 6 years of age should be made individually, taking compliance into consideration. The adjustment of formulation and dosing of medication during treatment is necessary in young children. Further research with larger groups of patients is needed to confirm our findings.

## 1. Introduction

A fixed-dose combination of sofosbuvir/ledipasvir (SOF/LDV) was shown to be highly effective, safe, and well tolerated in children infected with hepatitis C virus (HCV) aged 3 years and older [1,2,3]. Thus, based on the available results of clinical trials, SOF/LDV was approved for the treatment of chronic hepatitis C (CHC) by the European Medicines Agency and US Food and Drug Administration in adolescents aged 12–17 years (in 2017) and in children between 3 and 11 years of age (in 2020) [1,2,3]. However, real-life data on the efficacy and safety of SOF/LDV in pediatric patients are sparse [3,4,5,6]. In particular, observations in children under 12 years of age and in pediatric patients with comorbidities are lacking [6]. Thus, in this brief report, we aimed to present a case series report of the effects of SOF/LDV treatment in three patients aged 5 to 10 years suffering from comorbidities.

## 2. Materials and Methods

In this study, we report on children aged less than 12 years included in our real-life therapeutic program ‘Treatment of Polish Children with Chronic Hepatitis C Using Direct Acting Antivirals (POLAC Project)’ launched in our tertiary health care department for HCV-infected children (genotypes 1 and 4) from all Polish regions. The program is available courtesy of the donation of SOF/LDV by a pharmaceutical company. In total, between 2019 and 2021, 40 participants aged 5 to 17 years were included. The results of the treatment in the older age group (12 to 17 years) were described previously [4]. In this project, we included children with detectable HCV RNA for at least 6 months, irrespective of the stage of liver fibrosis or previous antiviral treatment. The duration of SOF/LDV treatment was 12 weeks unless the patient was infected with HCV genotype 1, had a history of previous ineffective interferon-based treatment and presented with cirrhosis [7]. All participants were prospectively followed every 4 weeks during the treatment and at the end of the therapy (EOT). The treatment was considered successful when the patient achieved a sustained virologic response 12 weeks after the end of treatment (SVR12). Laboratory testing was performed using commercially available laboratory kits, and nucleic acid testing for HCV RNA was performed using real-time polymerase chain reaction (RT–PCR method; Abbott m2000, with a lower limit of detection of 12 IU/mL). All of the patients and/or their parents/guardians gave written informed consent before their inclusion in the study.

## 3. Results

Among the participants included in the project, there were three children aged less than 12 years: a 10-year-old girl (Patient 1) and 5-year-old twins (a boy, Patient 2, and a girl, Patient 3). They started therapy in May and November 2021. All three children were infected vertically with genotype 1 HCV, and they were negative for HBs antigen, anti-HBc antibodies, and anti-HIV antibodies. The baseline clinical and laboratory characteristics of the patients are presented in Table 1. Patient 1 was treatment-experienced, and her liver stiffness measurement (LSM) at baseline was 7.5 kPa, corresponding to fibrosis F2 on the Metavir scale. No fibrosis (Metavir F0-1) was found in Patients 2 and 3. All patients suffered from comorbidities. In Patient 1, dural sinus thrombosis was diagnosed one year before SOF/LDV therapy, and she was treated with daltaparin. The twins were born prematurely in the 29th week of gestation. They were diagnosed with delayed psychomotor development, chronic kidney disease (with normal creatinine levels at baseline), renal hypodysplasia, and growth deficiency. In addition, Patient 2 had hypertension treated with ramipril. He also received trimethoprim/sulfamethoxazole to prevent a urinary tract infection. Before starting the treatment with SOF/LDV, possible drug interactions in Patients 1 and 2 were excluded using the HEP Drug Interaction Checker by the University of Liverpool (https://www.hep-druginteractions.org/checker, accessed on 28 September 2021).

All three patients qualified for 12 weeks of therapy with a fixed daily dose of SOF/LDV. Dosing and formula of SOF/LDV were adjusted to the children’s weight: Patient 1 received tablets containing 400/90 mg of SOF/LDV, whereas Patients 2 and 3 (weighting below 17 kg at baseline) received pellets containing 150/33.75 mg of SOF/LDV. After 4 weeks of treatment, due to the weight gain in Patients 2 and 3 to 17 kg, the SOF/LDV dose was increased to 200/45 mg. At this point, the alanine aminotransferase levels decreased in all patients to the normal range (below 40 IU/L), and in all cases, HCV RNA was undetectable (Table 1). At the EOT, as well as 12 weeks later, HCV RNA was still undetectable in all three cases, which confirmed that all patients achieved SVR12. Thus, treatment was considered successful, and HCV infection was eliminated. In addition, LSM in Patient 1 performed 12 weeks after the EOT revealed a significant decrease to 5.7 kPa (corresponding to F0-1 on the Metavir scale). Creatinine levels in Patients 2 and 3 were normal during the treatment and 12 weeks after EOT. No adverse events, including severe effects, were reported during SOF/LDV treatment by any patient.

## 4. Discussion

The results of clinical trials have shown that children with CHC can be safely treated with direct-acting antiviral agents (DAAs), with a similar efficacy to that observed in adults [3]. However, data on the efficacy, safety, and tolerability of SOF/LDV in specific groups of patients, such as children younger than 12 years or with comorbidities (e.g., chronic kidney disease), are lacking [6]. Few clinical trials and real-life studies have been performed on children aged 3–11 years infected mainly with genotype 4 HCV and treated with SOF/LDV [2,8,9,10,11,12,13]. They showed treatment efficacy ranging from 95 to 100% [2,8,9,10,11,12]. It was demonstrated that the efficacy in patients aged 3 to 6 years was lower, which indicates that patient age may influence therapy outcomes [3]. One possible reason for this may be the noncompliance in this age group resulting from difficulties in taking the oral medication, described as having a ‘bad taste’ [3,8]. Taking into consideration the natural history of CHC, including an extremely low risk of disease progression during the first years of life and an estimated 20–40% chance for spontaneous clearance within the first 6 years of life, the decision on starting treatment in children below 6 years of age should be made individually [3,13,14]. If the child is not able to swallow medications, it seems reasonable to postpone the therapy. Pellet formulations are more acceptable, but no syrups are available for children who have problems swallowing medications. In our report, we demonstrated that 12 weeks of treatment with a fixed-dose combination of SOF/LDV was effective in all three patients, safe, and well tolerated, with no compliance problems. However, our 5-year-old patients who received SOF/LDV pellets had a significant medical history with several comorbidities and were accustomed to swallowing medications. In addition, considering that the only curable disease in these patients was CHC, they would significantly benefit from the treatment and thus were included in the project. As they suffered from chronic kidney disease, their renal function was monitored during and after treatment, and no deterioration was observed. We also monitored their weight and body mass index as they presented with growth deficiency. Our recent findings had shown that treatment with SOF/LDV in children with CHC aged 10 to 17 years does not negatively influence patient growth [15]. In the case of all three patients aged 5 to 10 years, their BMI z scores at 12 weeks posttreatment were higher than those at baseline (Table 1). However, changes in the body weight of Patients 2 and 3 (from below to over 17 kg) during the treatment resulted in the need to adjust the medication dose.

CHC is a progressive disease. Our 10-year-old Patient 1 presented with F2 fibrosis at baseline. Our observations suggest that even 11% of teenagers with vertically infected CHC may develop significant fibrosis [16]. Interestingly, there was a significant improvement in her LSM at 12 weeks posttreatment to 5.7 kPa, correlating to F0-1 on the Metavir scale. This is consistent with our previous observations in teenagers that suggested the possibility of regression of liver fibrosis after treatment with SOF/LDV [17]. However, antiviral treatment should not be delayed, and the problem lies in the lack of availability of therapies for children with CHC in national therapeutic programs. In Poland, children receive treatment courtesy of drug donations from pharmaceutical companies and both commercial and non-commercial clinical trials.

Another issue would be to avoid HCV transmission from infected mothers to their children by treating pregnant women. However, as human data on the use of DAA during pregnancy are still lacking, early treatment of children with CHC remains the optimal strategy currently [18].

This study is limited as it was a case series report of only three patients. However, considering the low prevalence of young children diagnosed and treated for CHC, we hope that our experience with successful treatment of these children with comorbidities in a real-world setting will encourage other clinicians to use SOF/LDV in similar patients.

In conclusion, the treatment of CHC in children aged 5 to 10 years with comorbidities was safe and effective; however, decisions on starting treatment in children below 6 years of age should be made individually, taking compliance into consideration. The adjustment of formulation and dosing of medication during treatment is necessary in young children. Further research with larger groups of patients is needed to confirm our findings.

## Figures and Tables

**Table 1:** Clinical and laboratory characteristics of patients treated with sofosbuvir/ledipasvir.

Feature		Patient 1	Patient 2 *	Patient 3 *
Sex		Female	Male	Female
Age at start of the treatment		10 years 5 months	5 years 5 months	5 years 5 months
HCV genotype		1b	1a	1a
Mode of HCV infection		Vertical	Vertical	Vertical
BMI (kg/m^2^)/BMI z score	Start of LDV/SOF	18.1/0.52	15.5/0.17	15.5/0.16
	Posttreatment week 12	19.7/0.95	16.4/0.80	16.0/0.45
Previous anti-HCV treatment		Yes (Interferon plus ribavirin)	No	No
Comorbidities		Dural sinus thrombosis	Prematurity, delayed psychomotor development, chronic kidney disease, renal hypodysplasia, hypertension, growth deficiency	Prematurity, delayed psychomotor development, chronic kidney disease, renal hypodysplasia, growth deficiency
Concomitant medications		Dalteparin	Ramipril; thrimetoprim/ sulfamethoxazole	No
ALT (IU/mL)	Start of LDV/SOF	122	42	52
	4 weeks	28	16	35
	12 weeks	22	26	50
	Posttreatment week 12	17	16	16
HCV viral load (IU/mL)	Start of LDV/SOF	4.36 × 10^4^	3.8 × 10^6^	6.03 × 10^5^
	4 weeks	Undetectable	Undetectable	Undetectable
	12 weeks	Undetectable	Undetectable	Undetectable
	Posttreatment week 12	Undetectable	Undetectable	Undetectable
LSM (median; kPa/Metavir)	Start of LDV/SOF	7.5/F2	4.0/F0-F1	3.1/F0-F1
	Posttreatment week 12	5.7/F0-F1	4.3/F0-F1	4.9/F0-F1

ALT—alanine aminotransferase; BMI—body mass index; LDV/SOF—ledipasvir/sofosbuvir; LSM—liver stiffness measurement; * twins.

## Data Availability

The datasets used and analyzed during the current study are available from the corresponding author upon reasonable request.
